# Ultra-Processed Food Products and Obesity in Brazilian Households (2008–2009)

**DOI:** 10.1371/journal.pone.0092752

**Published:** 2014-03-25

**Authors:** Daniela Silva Canella, Renata Bertazzi Levy, Ana Paula Bortoletto Martins, Rafael Moreira Claro, Jean-Claude Moubarac, Larissa Galastri Baraldi, Geoffrey Cannon, Carlos Augusto Monteiro

**Affiliations:** 1 Departamento de Nutrição, Faculdade de Saúde Pública, Universidade de São Paulo, São Paulo, Brazil; 2 Núcleo de Pesquisas Epidemiológicas em Nutrição e Saúde, Faculdade de Saúde Pública, Universidade de São Paulo, São Paulo, Brazil; 3 Departamento de Medicina Preventiva, Faculdade de Medicina, Universidade de São Paulo, São Paulo, Brazil; 4 Departamento de Nutrição, Escola de Enfermagem, Universidade Federal de Minas Gerais, Belo Horizonte, Brazil; NIDDK/NIH, United States of America

## Abstract

**Background:**

Production and consumption of industrially processed food and drink products have risen in parallel with the global increase in overweight and obesity and related chronic non-communicable diseases. The objective of this study was to analyze the relationship between household availability of processed and ultra-processed products and the prevalence of excess weight (overweight plus obesity) and obesity in Brazil.

**Methods:**

The study was based on data from the 2008–2009 Household Budget Survey involving a probabilistic sample of 55,970 Brazilian households. The units of study were household aggregates (strata), geographically and socioeconomically homogeneous. Multiple linear regression models were used to assess the relationship between the availability of processed and ultra-processed products and the average of Body Mass Index (BMI) and the percentage of individuals with excess weight and obesity in the strata, controlling for potential confounders (socio-demographic characteristics, percentage of expenditure on eating out of home, and dietary energy other than that provided by processed and ultra-processed products). Predictive values for prevalence of excess weight and obesity were estimated according to quartiles of the household availability of dietary energy from processed and ultra-processed products.

**Results:**

The mean contribution of processed and ultra-processed products to total dietary energy availability ranged from 15.4% (lower quartile) to 39.4% (upper quartile). Adjusted linear regression coefficients indicated that household availability of ultra-processed products was positively associated with both the average BMI and the prevalence of excess weight and obesity, whereas processed products were not associated with these outcomes. In addition, people in the upper quartile of household consumption of ultra-processed products, compared with those in the lower quartile, were 37% more likely to be obese.

**Conclusion:**

Greater household availability of ultra-processed food products in Brazil is positively and independently associated with higher prevalence of excess weight and obesity in all age groups in this cross-sectional study.

## Introduction

The prevalence of obesity has reached alarming levels in almost all countries of the world [1], [2]. In Brazil, increasing rates of obesity have been documented by repeated national surveys conducted since the 1970 s with evidence of acceleration in the 2000 s in all age groups above 5 years old [3]. A nationwide surveillance system based on telephone interviews implemented in all state capitals of the country since 2006 indicates an annual increase of around one per cent in the prevalence of obesity and of excess weight (overweight plus obesity) among adults [4].

It is now commonly stated that the pandemic of obesity is driven by radical changes in the global food system, and in particular since the 1980 s by the increased production, availability, affordability and marketing of processed food and drink products [2], [5], [6]. International authorities now increasingly recognize that high levels of consumption of various specific types of processed food or drink products are associated with weight gain and associated chronic non-communicable diseases [7], [8].

Food processing as such has however been largely ignored in dietary recommendations, dietary assessments, and epidemiological studies. One sufficient reason for this has been the non-existence of clear definitions and classifications of processed foods [9], [10]. In recent years a classification of foodstuffs based on the extent, nature and purpose of food processing has been developed, and results based on the classification have been published. This divides foodstuffs into three groups. These are foods that are either fresh or minimally processed; processed culinary ingredients; and ready-to-consume food products, either processed or ultra-processed. Processed products are whole foods preserved with salt, sugar or oil or by other methods such as smoking or curing. Ultra-processed products are essentially industrial formulations mostly or entirely made from industrial ingredients, typically containing little or no whole foods. [11], [12].

Studies in different countries show that ready-to-consume food products (processed or ultra-processed) taken together as a group, when compared with foods combined with processed culinary ingredients as made into dishes and meals, on average are more energy-dense, are higher in total fat, saturated fats, sugars and salt, and are lower in protein and dietary fiber [13], [14]. Ultra-processed products in particular typically have properties that are conductive to overconsumption: they are often hyper-palatable and sold in large portion sizes; are durable and easy to transport and therefore liable to be consumed as snacks at any time and in almost any place; and are often marketed intensively and persuasively [15], [16], [17].

There is therefore reason to believe that high consumption of ready-to-consume food products in general, is a cause of weight gain, obesity and associated disorders and diseases [11], [16]. The only study so far conducted on the subject has reported an association between high consumption of these products and the occurrence of metabolic syndrome in adolescents from a medium-sized Brazilian city [18]. The association with other health outcomes at this time remains unknown.

Tracking and understanding the association between ready-to-consume food products and obesity, and the implications, is crucial. In Canada, between 1938 and 2011, the share of these products as a percentage of dietary energy rose from 28.7% to 61.7% [19]. In Brazil the contribution of dietary energy from these products has also risen: in metropolitan areas from 20.3% in 1987–8 to 32.1% in -2008–9; and nationality from 23.0% in 2002–3 to 27.8 in 2008–9. They continue to displace foods and processed culinary ingredients used together to make freshly prepared meals [20]. In the same time period, the prevalence of obesity has also increased in Brazil [3]. The objective of this study has been to analyze the relationship between household availability of processed and ultra-processed products, separately and together, and the prevalence of excess weight (overweight plus obesity) and obesity in Brazil.

## Methods

### Data Source and Sample

All the data come from the 2008–2009 Household Budget Survey (HBS), conducted by the Brazilian Institute of Geography and Statistics on a probabilistic sample of 55,970 households [21].

The 2008–2009 HBS employed a complex clustered sampling procedure, first selecting census tracts and then selecting households within those tracts. The selection of census tracts was preceded by an examination of the tracts of the Master Sample of Household Surveys or Common Sample (containing the pool of the 12,800 tracts of the country) to obtain strata of households with high geographic and socioeconomic homogeneity. The geographic locations of tracts (region, state, capital city or other, urban or rural) and the years of schooling of the heads of households in the sector were considered, and 550 strata of households that were geographically and socioeconomically homogeneous were selected. The number of tracts selected from each stratum was proportional to the total. The number of sectors randomly selected from each stratum was proportional to the total number of households in the stratum. Next, households were selected in each tract by random sampling without reposition. Interviews were distributed uniformly in each selected stratum during the four quarters of the study to reproduce seasonal variations in purchases of food and other products [21].

### Data Collection

The main information taken from the 2008–2009 HBS included the household purchase of foods and drinks for home consumption and the weight and height of all household members.

The purchase records of all foods and drinks for home consumption (approximately 850,000) were recorded in a specially designed booklet by the household members (or by the interviewer, when necessary) over a period of seven consecutive days [22]. Due to the relatively short reference period employed for the recording of the food expenditure in each household, it was decided to use the 550 sample strata as the study unit, for which the pattern of annual food purchases could be more accurately calculated. The mean number of households studied within each stratum was 101.8, ranging from eight to 796 households.

Weight and height of all people residing at the household (n = 190.159) were measured by trained researchers using standard techniques, and recorded in specific questionnaires, along with characteristics of households and their members. Weight was measured using portable electronic scales with a maximum capacity of 150 kilograms (kg), and graduations of 100 grams (g). The value obtained was recorded in kilograms. Height was expressed in centimeters (cm) using recumbent length as the measure in children aged between zero and 23 months and stature in individuals aged 24 months or older. In order to measure length, infant anthropometers were used with a capacity of up to 105 cm and a scale in millimeters, whereas stature was measured using portable stadiometers with a 200 cm-long retractable tape measure, accurate to the nearest 0.1 cm. Upon completion of data collection, imputation procedures were applied to deal with non-responses or erroneous responses associated with values rejected at the critical review stage [3].

### Classification of Purchased Food Items

All food items purchased by households, after the exclusion of non-edible parts [23], were converted into energy using the Brazilian Food Composition Table (TACO) [24] or as necessary the US official nutrient database for standard reference [25]. The quantity of each purchased food in each household stratum was expressed in daily kilocalories (kcal) *per capita.*

Subsequently, the food items were classified into three groups, according to the nature, extent and purpose of industrial processing used in their manufacture [11], [12]. The first group is of foods, either fresh or minimally processed. Examples are grains (also known as cereals), and roots and tubers; legumes (pulses); fruits and vegetables; nuts and seeds; meat, fish, poultry and eggs; milk and natural yogurt. The second group is of processed culinary ingredients used with foods in the preparation of dishes. These are substances extracted from whole foods. Examples are flours and starches; oils and sugars; and salt (extracted from nature). The third group, the main subject of this study, is of ready-to-consume products. These are either processed or ultra-processed.

Processed products are made from foods with the addition of substances such as salt, sugar or oil, and the use of processes such as smoking or curing. Examples include canned or bottled vegetables and legumes preserved in brine; fruits preserved in syrup; tinned fish preserved in oil or salted and smoked; salted and smoked meats; and cheese.

Ultra-processed products are formulated predominantly or entirely from industrial ingredients, and typically contain little or no whole food. They often contain preservatives and cosmetic and other additives, and may also contain synthetic vitamins and minerals. Examples include: cake mixes, ‘energy’ bars; ‘instant’ packaged soups and noodles; many types of sweetened breads and buns, cakes, biscuits, pastries and desserts; chips (crisps); and very many other types of sweet, fatty or salty snack products; sugared milk and fruit drinks, soft drinks and ‘energy’ drinks; pre-prepared meat, fish, vegetable or cheese dishes, pizza and pasta dishes, burgers, French fries (chips), and poultry and fish ‘nuggets’ or ‘sticks’ (‘fingers’); bread and other cereal products; hot dogs and other products made with scraps or remnants of meat; preserves (jams), sauces, meat, yeast and other extracts; ice-cream, chocolates, cookies (biscuits), candies (confectionery); margarines; canned or dehydrated soups; and infant formulas, follow-on milks and baby products.

### Indicators of Obesity

We calculated the values of body mass index (BMI), for adults and elderly, and BMI-for-age, for children and adolescents, based on weight and height measurements taken. These values were expressed in Z-scores and were used for classification of the nutritional status, following the recommendations proposed by the World Health Organization for each age group [26], [27], [28].

Three different indicators of obesity were studied: the mean BMI (in Z-score), the prevalence of excess weight, defined as the percentage of people with BMI above 25 kg/m^2^ for adults or above +2 Z-score for children under 5 years and +1 Z-score for children and adolescents (5 to 19 years), and the prevalence of obesity, defined as the percentage of people with BMI above 30 kg/m^2^ for adults or above +3 Z-score for children under 5 years and +2 Z-score for children and adolescents (5 to 19 years). All indicators were calculated for each stratum (our study unit), including prevalence of excess weight and obesity, and these outcomes were used in the linear regression models.

### Data Analysis

Initially, the amounts of processed and ultra-processed products were estimated. The mean values of excess weight and obesity prevalence and BMI were calculated according to quartiles of the dietary energy (expressed as calories) of the processed and ultra-processed products as a proportion of the total purchased.

Multiple linear regression models were used to assess the association between the availability of processed and ultra-processed products (expressed in quartiles of calories), first separated, and each one of the indicators of obesity (outcomes). We included in the models socio-demographic variables frequently associated with food consumption and nutritional status, such as region, setting, income, gender and age, these last expressed as proportion of women, elderly and children in the stratum. Furthermore, we included other confounding variables, such as percentage of expenditure on eating out of home and complementary dietary energy (derived from foods and processed culinary ingredients). These were variables available in the database used.

Based on these models, the expected values (values predicted by model) were calculated for the excess weight and obesity prevalence and for average BMI, according to the quartiles of dietary energy from processed and ultra-processed products, adjusted for the mean values of the confounding variables included in the models.

All analyses were carried out using the statistics package Stata/SE version 12.1 (Stata Corp., College Station, USA), considering the effects of complex sampling of the 2008–2009 HBS and enabling the extrapolation of the results for the entire Brazilian population.

### Ethical Aspects

The present study used secondary data (2008–2009 HBS) collected by the IBGE and available for public online consultation. The information contained in the database is confidential since specific data about each household such as identification of the household members, address and telephone are excluded.

## Results

The average daily dietary energy household availability was 1581 kcal/person. Of this, processed products contributed 37 kcal (2.4%) and ultra-processed products contributed 386 kcal (25.5%).

Table 1 shows that as the contribution of processed and ultra-processed products, as a group, to dietary energy increased (from 15.4% to 39.4%) from the lower to the upper quartiles the prevalence of excess weight and obesity also increased (from 34.1% to 43.9%, and from 9.8% to 13.1%, respectively). We first assessed the association between each of the three outcomes and the dietary energy of processed products and ultra-processed products separately. The results showed that ultra-processed products were associated with the average BMI and with prevalence of both excess weight and obesity in the adjusted models, whereas processed products were not. Considering this, results in Table 2 and Table 3 are presented for ultra-processed products only. Table 2 shows the results of linear regression models for the association between dietary energy from ultra-processed products, and excess weight and obesity. Both crude and socio-demographic-adjusted regression coefficients show a positive and statistically significant association. The variables that are most responsible for the changes in the estimates from the crude to the adjusted models are income and setting (urban or rural). Additional adjustment of dietary energy other than from ultra-processed products made no significant difference. The residuals analysis of the linear regression models indicated a reasonable fit in the models (data not shown).

**Table 1:** Indicators of obesity among all age-groups according to the share of processed and ultra-processed food products in total household food availability (Brazil, 2008–2009).

	Obesity indicator		
	Mean BMI (Z score)	Prevalence of excess weight (%)^1^	Prevalence of obesity (%)^1^
Quartiles of the contribution of processed and ultra-processed products (% of total dietary energy)	Mean (SE)	Mean (SE)	Mean (SE)
1^st^ (1. 6%–21.8%)	0.53 (0.02)	34.1 (0.6)	9.8 (0.3)
2^nd^ (21.8%–28.3%)	0.68 (0.02)	39.6 (0.6)	12.3 (0.3)
3^rd^ (28.5%–34.8%)	0.81 (0.02)	43.8 (0.6)	14.1 (0.4)
4^th^ (34.8%–54.9%)	0.81 (0.02)*	43.9 (0.6)*	13.1 (0.5)*

1 Classification follows recommendations of the World Health Organization for each age group [26], [27], [28].

*p<0.05 for linear regression across quartiles of dietary energy contribution of processed and ultra-processed products.

**Table 2:** Results from multiple linear regression models for the association between household availability of ultra-processed food products (kcal/person/day) and obesity indicators (Brazil, 2008–2009).

Obesity indicator	Quartiles of availability of ultra-processed products	*Crude Coefficient (95%CI) (model 1)*	*Adjusted coefficient (95%CI) (model 2)* 2	*Adjusted coefficient (95%CI) (model 3)* 3
**Mean BMI (Z score)**	1^st^	Ref	Ref	Ref
	2^nd^	0.16 (0.10; 0.21)	0.08 (0.04; 0.12)	0.10 (0.06; 0.14)
	3^rd^	0.20 (0.15; 0.26)	0.10 (0.06; 0.15)	0.13 (0.08; 0.18)
	4^th^	0.33 (0.28; 0.38)*	0.15 (0.10; 0.21)*	0.19 (0.14; 0.25)*
**Prevalence of excess weight (%)** 1	1^st^	Ref	Ref	Ref
	2^nd^	5.56 (3.75; 7.46)	2.81 (1.39; 4.23)	3.25 (1.85; 4.66)
	3^rd^	7.23 (5.48; 8.98)	3.40 (1.89; 4.92)	4.20 (2.61; 5.79)
	4^th^	11.52 (9.66; 13.38)*	5.09 (3.17; 7.00)*	6.27 (4.15; 8.39)*
**Prevalence of obesity (%)** 1	1^st^	Ref	Ref	Ref
	2^nd^	2.51 (1.48; 3.53)	2.10 (1.23; 2. 98)	2.28 (1.49; 3.07)
	3^rd^	3.16 (2.34; 3.97)	2.15 (1.29; 3.01)	2.42 (1.48; 3.35)
	4^th^	4.88 (3.70; 6.05)*	3.27 (2.07; 4.47)*	3.72 (2.50; 4.94)*

1 Classification follows recommendations of the World Health Organization for each age group [26], [27], [28].

2 Adjusted by log of income, proportion of women in stratum, proportion of elderly in stratum, proportion of children in stratum, setting, region, and percentage of expenditure on eating out of home (Model 2).

3 Model 2 plus adjustment for complementary calories, including calories of processed food products (Model 3).

* Linear trend across quartiles of calories ultra-processed products was significant (p<0.001).

**Table 3:** Predictive values for obesity indicators according to the household availability of ultra-processed food products (Kcal/person/day) (Brazil, 2008–2009).

	Obesity indicator		
Availability of ultra-processed products (mean values according to quartiles)	Mean BMI(Z score)^2^	Prevalence of excessweight (%)^1^^,^ 2	Prevalence of obesity (%)^1^^,^ 2
1^st^ (220.0 kcal)	0.56	35.6	9.9
2^nd^ (345.6 kcal)	0.66	38.7	12.0
3^rd^ (422.0 kcal)	0.69	39.6	12.3
4^th^ (564.3 kcal)	0.75	41.7	13.6

1 Classification follows recommendations of the World Health Organization for each age group [26], [27], [28].

2 Adjusted indicators correspond to predicted values yielded by Model 3 (adjusted by log of income, proportion of women in stratum, proportion of elderly in stratum, proportion of children in stratum, setting, region, percentage of expenditure on eating out of home, and for complementary calories, including calories of processed food products), set for the mean value of the confounding variables.

Table 3 shows the predictive adjusted values of average BMI and the prevalence of excess weight and obesity according to the quartiles of household availability of ultra-processed products. People living in household strata belonging to the upper quartile (average 564 kcal) of consumption of ultra-processed products, compared with people in the lower quartile (average 220 kcal), were 37.4% more likely to be obese (from 9.9% to 13.6%).

## Discussion

We believe that this study is the first to examine the relationship between consumption of ultra-processed products and obesity. Using a national representative sample of the Brazilian population of all age groups, a positive and independent association has been found between the household availability of ultra-processed products and obesity.

These results are also relevant globally. Ultra-processed products dominate food supplies of many high-income countries, and production and consumption of these products is now rapidly increasing in middle-income countries and settings [29].

In this study we used data of HBS related to food purchase. We believe that our data are a reasonable estimative of intake, because previous studies indicate considerable agreement between data from HBS and individual food consumption surveys [30], [31]. Foods and products bought and consumed out of home were not included in the survey. To account for potential bias, the percentage of food expenditure allocated to food consumed out of home was considered. This variable adjusted for income was taken as a “proxy” for dietary energy consumed out of home, which in Brazil, at the time has been estimated at 18% of dietary energy [32]. Our study also does not take into account household food wastage. However, ultra-processed products are usually durable and have long shelf lives, and therefore generate little or no waste. So our data most probably underestimate the availability of ultra-processed products in Brazil.

Physical activity and also smoking are not usually assessed in household budget surveys and so could not be included them as potential confounders for the association between consumption of ultra-processed products and obesity. However, previous studies in Brazil have found that physical activity patterns are strongly dependent on variables which were effectively controlled in our analyses, including gender, age, family income, urban or rural settings and the country’s five regions [33], [34]. Also, the nationwide surveillance system for chronic diseases has shown that education (a “proxy” for income) and gender are related to smoking, and both these variables were included in the analyses [4]. In any case, as usual in observational studies, residual confounding can not be discarded.

Due to the inclusion of all age groups in the analyses and the lower predictive value of BMI in the assessment of obesity in the elderly, we have conducted a sensitivity analysis excluding strata with more than 20% of individuals with 65 years or plus. Similar analysis was conducted considering only individuals older than 20 years. No changes in the magnitude or statistical significance of coefficients were found. Finally, an additional sensitivity analysis was done with the exclusion of strata with less than 30 households (2.55%) but this not changed the results and conclusions of the study and for these reasons, we used the original number of strata (n = 550) to the analyses.

Furthermore, residual confounding due to imperfect measurement of income is also possible since income was reported by the families. We believe this problem has been attenuated because income data include all sources of income from all household members and were collected by trained interviewers with standardized and carefully detailed questionnaires.

Our findings are consistent with the few studies that have examined the impact of food processing, or products that can be classified as ultra-processed, on obesity.

In Guatemala, one study conducted using a representative sample of households has investigated the association between the prevalence of overweight/obesity and household food expenditure on processed food products. Using a somewhat different classification from ours, this study reported that a 10% increase in the proportion of “partially processed” and “highly processed” foods in total food expenditure was associated with an increase in the mean BMI of around 4% [35]. Most items included in the “partially processed” and “highly processed” groups belong to our group of ready-to-consume products.

In the US, data from three cohorts (from 12 to 20 years) has reported an association between weight gain and increased consumption of various ultra-processed products, including French fries (also known as chips), potato chips (crisps), sweetened drinks, and processed meats, whereas several fresh and minimally processed foods were considered protective against weight gain [36].

Other prospective studies have confirmed an association between specific ready-to-consume products and weight gain, as well as other negative health outcomes. A 15-year prospective study, examining the consumption of fast-food snacks by North American young adults, has shown that changes in the frequency of weekly consumption of these products was directly associated with changes in body weight [37]. Another study conducted in five European countries for 5.5 years has found that a daily rise of 100 kcal in consumption of ultra-processed products such as white bread, processed meats, and soft drinks was positively associated with an increase in abdominal adiposity [38].

Finally, regular consumption of sweetened soft drinks is now generally agreed to increase incidence of overweight and obesity, and is also associated with increased incidence of disorders and diseases such as type-2 diabetes, cardiovascular diseases, hypertension, inflammation, atherogenic dyslipidemia, hyperuricaemia, gout, gall stones and renal diseases [39], [40], [41].

We suggest that the association with obesity found in our study is a result of many characteristics of ultra-processed products. These include their nutritional profile (as a group in general they are more energy-dense, and more fatty and more sugary, than the combination of foods and culinary ingredients made into freshly prepared meals) [13], [14]. As a group they also stimulate overconsumption (by their hyper-palatability, large portion sizes, convenience, and aggressive and persuasive marketing strategies) and the way of eating (they can be consumed at any time and in almost any place) [15], [16], [17]. The absence of association between processed food products and obesity is probably due to these characteristics, unique to ultra-processed products, rather than to their nutritional profile. If the findings of this study are supported by findings from other countries, they have important implications for public health policy. In recent decades very large including transnational food and drink corporations, most of whose products are ultra-processed, have rapidly become much more prominent [10], [42].

This study in Brazil shows that increased consumption of ultra-processed food products is correlated with increased prevalence of excess weight and obesity. This we believe is because of the nature of these products and their intrinsic characteristics. We suggest that prevalence of excess weight and obesity can be controlled only if the production and consumption particularly of ultra-processed products is controlled and reduced.
